# Genome-Wide Identification of M35 Family Metalloproteases in *Rhizoctonia cerealis* and Functional Analysis of RcMEP2 as a Virulence Factor during the Fungal Infection to Wheat

**DOI:** 10.3390/ijms21082984

**Published:** 2020-04-23

**Authors:** Lijun Pan, Shengxian Wen, Jinfeng Yu, Lin Lu, Xiuliang Zhu, Zengyan Zhang

**Affiliations:** 1 Institute of Crop Sciences, National Key Facility for Crop Gene Resources and Genetic Improvement, Chinese Academy of Agricultural Sciences, Beijing 100081, China; plj7512@163.com (L.P.); lulin@caas.cn (L.L.); zhuxiuliang@caas.cn (X.Z.); 2 College of Agriculture, Hunan Agricultural University, Changsha 410128, China; wsx8725@hunau.net; 3 College of Plant Protection, Shandong Agricultural University; Taian 271018, China; jfyu@sdau.edu.cn

**Keywords:** cell death, M35 metalloprotease, reactive oxygen species, *Rhizoctonia cerealis*, virulence, wheat (*Triticum aestivum L*.)

## Abstract

*Rhizoctonia cerealis* is the causal pathogen of the devastating disease, sharp eyespot, of the important crop wheat (*Triticum aestivum L*.). In phytopathogenic fungi, several M36 metalloproteases have been implicated in virulence, but pathogenesis roles of M35 family metalloproteases are largely unknown. Here, we identified four M35 family metalloproteases from *R. cerealis* genome, designated RcMEP2–RcMEP5, measured their transcriptional profiles, and investigated RcMEP2 function. RcMEP2-RcMEP5 are predicted as secreted metalloproteases since each protein sequence contains a signal peptide and an M35 domain that includes two characteristic motifs HEXXE and GTXDXXYG. Transcription levels of *RcMEP2-RcMEP5* markedly elevated during the fungus infection to wheat, among which *RcMEP2* expressed with the highest level. Functional dissection indicated that RcMEP2 and its M35 domain could trigger H_2_O_2_ rapidly-excessive accumulation, induce cell death, and inhibit expression of host chitinases. This consequently enhanced the susceptibility of wheat to *R. cerealis* and the predicated signal peptide of RcMEP2 functions required for secretion and cell death-induction. These results demonstrate that RcMEP2 is a virulence factor and that its M35 domain and signal peptide are necessary for the virulence role of RcMEP2. This study facilitates a better understanding of the pathogenesis mechanism of metalloproteases in phytopathogens including *R. cerealis*.

## 1. Introduction 

Wheat (*Triticum aestivum*) is necessary for food security since it is one of the most important staple food crops worldwide [1]. Sharp eyespot is a destructive disease of wheat that can severely reduce yields (∼10–40%) of wheat in many regions of Asia, Oceania, Europe, North America, and Africa [2,3]. The necrotrophic fungus *Rhizoctonia cerealis* van der Hoeven, belonging to the binucleate *Rhizoctonia* subgroup AG-D I [4], is the major causal agent of sharp eyespot in wheat, barley, oats, and rye [5,6]. Since the late 1990s, China has become the largest epidemic region where more than 6.67 million ha of wheat plants can be infected by *R. cerealis* annually [7,8]. In addition, *R. cerealis* can also infect sugar beet, cotton, potato, several legumes, and turfgrasses, leading to root rot disease and yellow patch [9,10]. To improve using host-inducing gene silencing strategy in the resistance of wheat and other plants to *R. cerealis*, it is necessary to explore and further study the effectors or virulence factors of the fungal pathogen. Recently, we completed the genome sequencing of the *R. cerealis* strain Rc207 and finished genome de novo assembling and annotation, and completed time-course-infected transcriptome sequencing (Zengyan Zhang′s lab, unpublished data), which establishes databases for further study. In addition, our recent papers showed that by means of the purified proteins infiltrated into leaves of wheat and tobacco, a cutinase RcCUT1 and a xylanase RcXYN1 functioned as effectors in *R. cerealis* infection [11,12]. However, the functional roles of several hundred candidate effectors of *R. cerealis* remain largely unknown. 

Microbial pathogens have developed various strategies to infect their hosts and to cause diseases. Plant pathogenic fungi have been classified as biotrophs, hemi-biotrophs, and necrotrophs according to their lifestyles. Biotrophic pathogens have to obtain nutrients from living host cells and tissues, and thus they often secrete limited amounts of cell wall-degrading enzymes and effectors to suppress the host immune system [13]. In the infected plants, excess generation of reactive oxygen species (ROS) can promote plant cell death and is believed to arrest the growth of biotrophic pathogens [14,15]. In contrast, necrotrophic pathogens thrive on the dead host tissues that are killed before or during the microbial colonization. Necrotrophic fungi often secrete phytotoxic secondary metabolites and peptides, and generate ROS, all which can induce host-plant cell death [16,17]. Hemi-biotrophic pathogens, having a biotrophic phase at the early infection stage and a necrotrophic phase at the late infection stage, generate toxins and kill the host cells only at the late stage and finish their life cycle on the dead host tissues [15]. 

Metalloproteases include a diverse family of metal ion-dependent proteases and distribute widely in a broad diversity of pathogens [17,18]. Among them, protein sequences of zinc ion-dependent metalloproteases all contain the common HEXXH motif, in which two histidine (H) residues function as the first and the second zinc ligand, respectively [19,20,21]. The deuterolysin (M35) and the fungalysin (M36) families are the major types of zinc metalloproteinases secreted by pathogenic fungi [22]. These two families of metalloproteinases share little similarity with each other, with the exception of the HEXXH motif. M35 family proteases have both the HEXXH motif and another motif GTXDXXYG at the C-terminal, in which the D (asparagine) acts as the third zinc ligand [23]. Metalloproteinases have been shown to be important effectors or/and virulence factors of pathogenic bacterium and fungi with different lifestyles [24,25,26,27,28,29,30]. For instance, a zinc metalloprotease Avr-Pita of rice blast fungus *Magnaporthe oryzae* has been demonstrated to function as an effector and to interact with Pi-ta, a cytoplasmic resistance gene-encoding protein of rice [26]. In the pathogenic fungus *Colletotrichum graminicola* of the maize anthracnose disease, the M36 metalloprotease Cgfl has been demonstrated to act as an effector to enhance virulence [27]. However, functional roles of M35 family metalloproteinases in plant pathogenic fungi are largely unknown. 

In this study, we identified four M35 family metalloprotease genes from the assembled *R. cerealis* Rc207 genome sequences (unpublished data), analyzed their genes and protein sequences as well as phylogenetic relationship, and examined their transcriptional profiles in time-course during the fungal infection to wheat. We further investigated the functional role of the M35 family metalloprotease RcMEP2 and its signal peptide and M35 domain in the fungal pathogenesis to wheat, as RcMEP2 transcription is the most highly upregulated among the four genes during fungal infection. The results indicated that RcMEP2 acted as a virulence factor through triggering rapidly-excessive accumulation of ROS and inducing cell death and inhibiting the expression of chitinases’ genes in the treated wheat. Both its signal peptide and M35 domain of RcMEP2 are necessary for the virulence role of this metalloprotease. 

## 2. Results

### 2.1. Global Characterization of the M35 Family Metalloproteases in R. cerealis Genome

**Figure 1:**
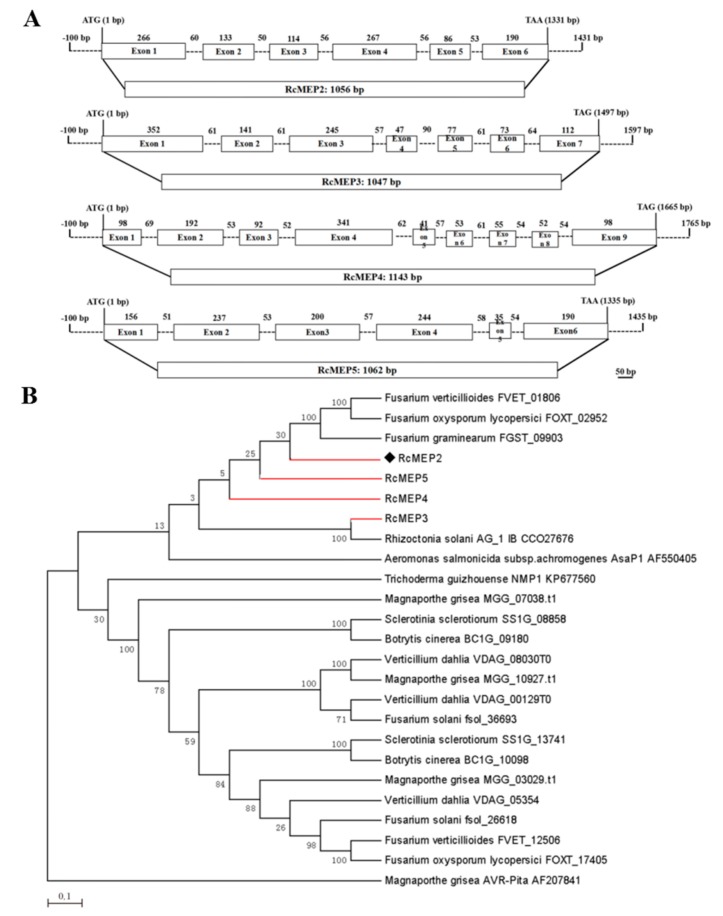
Gene structures of RcMEP2-RcMEP5, four M35 metalloproteases in *Rhizoctonia cerealis*, and their phylogenetic relationships. (**A**) Exons and introns are indicated by boxes and dash lines, respectively. (**B**) Phylogenetic relationship among RcMEP2-RcMEP5 and 21 other M35 family metalloproteases from other pathogenic fungi and the fish pathogenic bacterium *Aeromonas salmonicida* subsp. *Achromogenes*. The phylogenetic tree was constructed by Mega 7.0 using neighbor-joining (parameters: 1000 bootstraps).

Four M35 domain-containing deuterolysin/M35 metalloproteases were identified in the proteome derived from the *R. cerealis* Rc207 genome (unpublished) by means of searchinghomologous metalloprotease proteins with the MEROPS (The Peptidases database). They were designated as RcMEP2, RcMEP3, RcMEP4, and RcMEP5, respectively. These metalloprotease genes *RcMEP2-RcMEP5* have five to eight introns with Open Reading Frames (ORFs) ranging from 1047 to 1143 bp (Figure 1A). RcMEP3 was the smallest and comprised of 348 amino acid (AA) residues, RcMEP2 consisted of 351 AA, RcMEP5 included 353 AA, and RcMEP4 was the largest and composed of 380 AA residues. Based on signal peptide prediction, all of the four metalloproteases RcMEP2-RcMEP5 each include a signal peptide and thus were predicted as secreted proteins. Protein sequence analysis indicated that RcMEP2-RcMEP5 each had the two conserved motifs HEXXE and GTXDXXYG that are characteristic and important for the enzyme activity of M35 (deuterolysin) metalloproteases. To understand the evolutionary relationship among RcMEP2-RcMEP5 and 21 other M35 family metalloproteases from other phytopathogenic fungi and *Aeromonas salmonicida* subsp. *Achromogenes,* a bacterial pathogen of fishes, a phylogenetic tree was constructed using the neighbor-joining phylogeny method [31]. The resulting phylogenetic tree showed that RcMEP2-RcMEP5 were clustered into the same clade with four other M35 metalloproteases from four other plant pathogenic fungi (three *Fusarium* species and *R. oryzae*) and the known-functional M35 metalloprotease AsaP1 from the fish pathogenic bacterium *A. salmonicida* subsp. *Achromogenes*, although RcMEP2-RcMEP5 were utmost to AsaP1 (Figure 1B).

### 2.2. Transcriptional Profiles of RcMEP2-RcMEP5 during the Fungus Infection to Wheat

**Figure 2:**
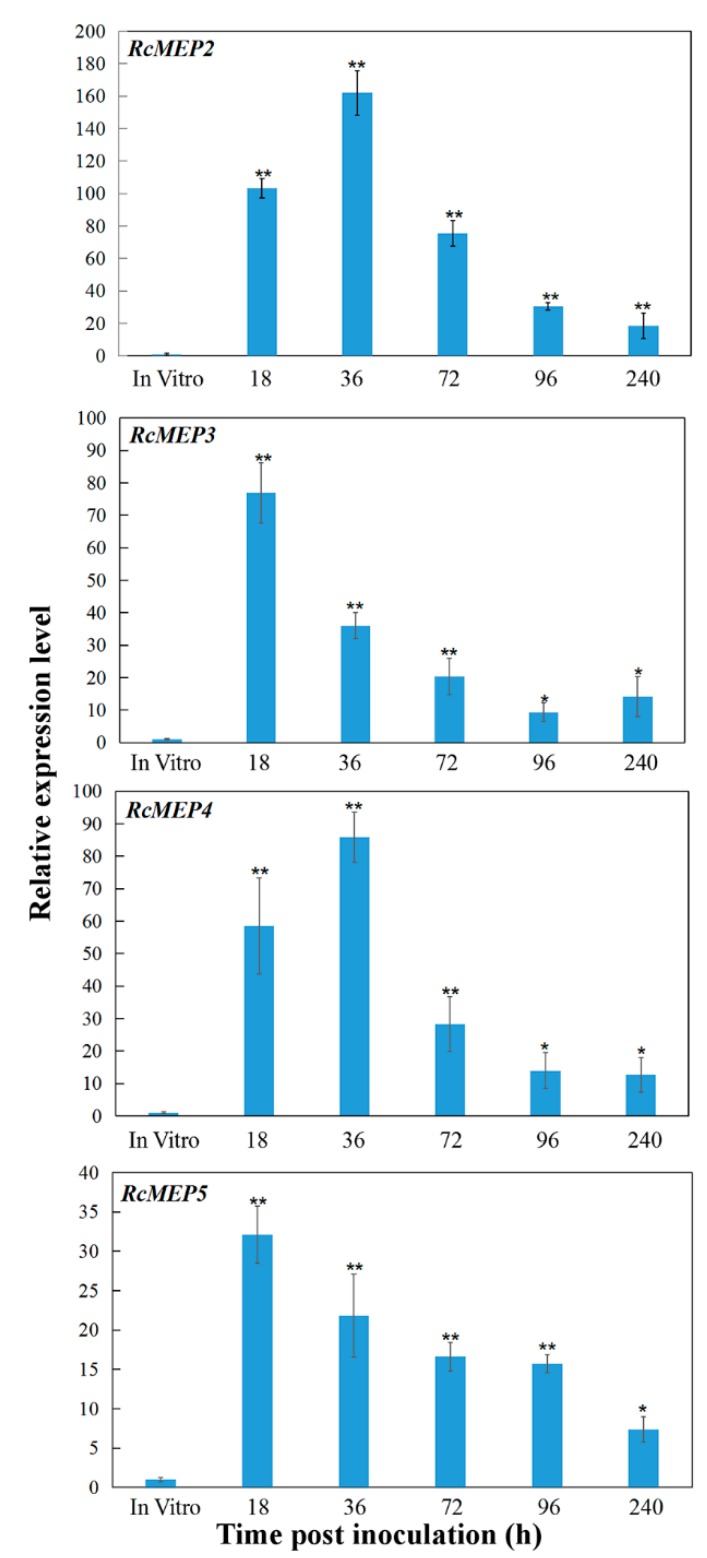
Transcriptional profiles of *RcMEP2-RcMEP5* genes in *Rhizoctonia cerealis* during the infection to wheat. The *R. cerealis Actin* gene was used as an internal control to normalize the data. The relative transcriptional abundances of *RcMEP2-RcMEP5* were examined during the infection process to wheat stems/sheaths, including five different infection time points (18, 36, 72, 96, and 240 h post inoculation (hpi)) and compared to in vitro culturing. Error bars were calculated based on three replicates. Asterisks indicate significant difference (** *p* < 0.01 or * *p* < 0.05, *t*-test) between the pathogen-inoculated sample and in vitro culturing.

The transcriptional profiles of *RcMEP2, RcMEP3, RcMEP4*, and *RcMEP5* were examined during the infection process to wheat basal sheaths/stems, including five different inoculation time points (18, 36, 72, 96, and 240 h post inoculation (hpi)) and in vitro culturing hypha. Quantitative RT-PCR (qRT-PCR) analyses indicated that during in vitro culturing, *RcMEP2, RcMEP3, RcMEP4*, and *RcMEP5* displayed a very low transcriptional level set as 1, respectively. In comparison with that in vitro culturing, transcriptional levels of *RcMEP2, RcMEP3, RcMEP4,* and *RcMEP5* were significantly upregulated (~10–162 times) during the infection process (at all time points) and reached peaks (~32- to 162-fold) at early infection stages (18–36 hpi) (Figure 2), implying that these four metalloproteases might play an important role in pathogenesis of the fungus infection to wheat. However, the induction degrees of these four metalloprotease genes were different during the infection process. Among them, the transcriptional induction of *RcMEP2* was the highest at all the tested infection time points and peaked at 36 hpi with ~162-fold over that in vitro culturing. The transcriptional induction degrees of RcMEP3 and RcMEP4 were in the middle, and their inductions peaked at 18 or 36 hpi with 78- or 85-fold over in vitro culturing (Figure 2). The transcriptional induction of RcMEP5 was the least and peaked at 18 hpi with ~32-fold over in vitro culturing (Figure 2). Based on these data and previous papers [27,28], we suspected that RcMEP2 might play an important virulence role in the fungal infection to wheat, and thus we further investigated its functional role.

### 2.3. RcMEP2 and Its M35 Domain-Containing Peptides Induce Cell Death in Infiltrated Plant Leaves 

**Figure 3:**
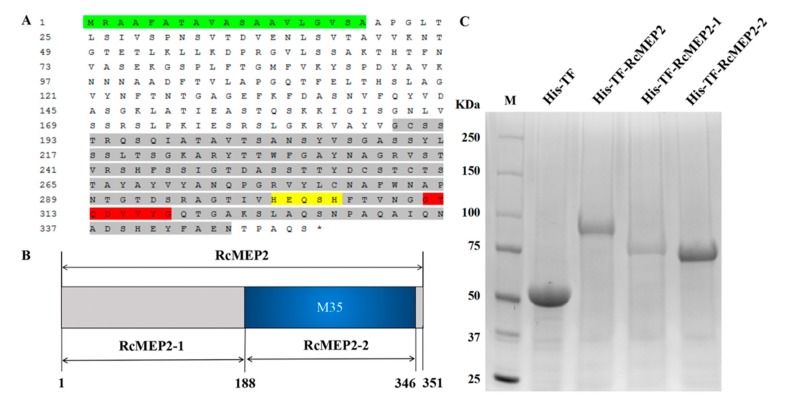
Protein structure of RcMEP2 and heterologous expression of RcMEP2 and its M35-containing/deleting mutants. (**A**) The deduced amino acid sequence of RcMEP2. Green part represents the signal peptide, yellow highlight indicates the HEQSH motif, the red indicates another motif GTQDVVYG, the M35 domain is marked by the grey. (**B**) Schematic view of the RcMEP2 and M35-containing/deleting domain proteins that are heterologously-expressed in *Escherichia* *coli* cells. (**C**) Sodium dodecyl sulphate-polyacrylamide gel electrophoresis (SDS-PAGE) patterns of the heterologous-expressing and purified proteins of His-TF-RcMEP2, His-TF-RcMEP2-1 (the M35 domain-deleting peptide), and His-TF-RcMEP2-2 (the M35 domain-containing peptide).

The protein sequence analysis showed that the deduced RcMEP2 protein comprised 351 amino acid (AA) residues (Figure 3A), with molecular weight 36.7 KD. As shown in Figure 3A, SignalP4.0 analysis and NCBI (National Center For Biotechnology Information) BLASTP (blast protein) results indicated that the RcMEP2 protein contained a signal peptide (located at no. 1 to 19 AA residues) and an M35 metalloprotease domain (at no. 189 to 346 AAs). Further analysis revealed that the M35 domain of RcMEP2 possessed the active site motifs HEQSH (at 301–305 AAs) and GTQDVVYG (at 311–318 AAs) that are characteristic to M35 metalloproteases (Figure 3A). These results suggested that RcMEP2 should be a metalloprotease belonging to the M35 family. In order to investigate whether the RcMEP2 protein has a virulence role and whether its M35 domain is required for the function of RcMEP2, we constructed three recombinant protein expressing vectors, including pHis-TF-RcMEP2 (expressing the full RcMEP2 protein), pHis-TF-RcMEP2-1 (expressing no. 1–189 AA residues of RcMEP2, the M35 domain-deleting peptide), and pHis-TF-RcMEP2-2 (expressing no. 190–351 AAs of RcMEP2, the M35 domain-containing peptide) (Figure 3B). Subsequently, the His-TF-RcMEP2, His-TF-RcMEP2-1, and His-TF-RcMEP2-2 recombinant proteins were highly expressed in *Escherichia* *coli*, respectively. After purification, these His-TF-RcMEP2, His-TF-RcMEP2-1, and His-TF-RcMEP2-2 proteins were checked using sodium dodecyl sulphate-polyacrylamide gel electrophoresis (SDS-PAGE) (Figure 3C).

**Figure 4:**
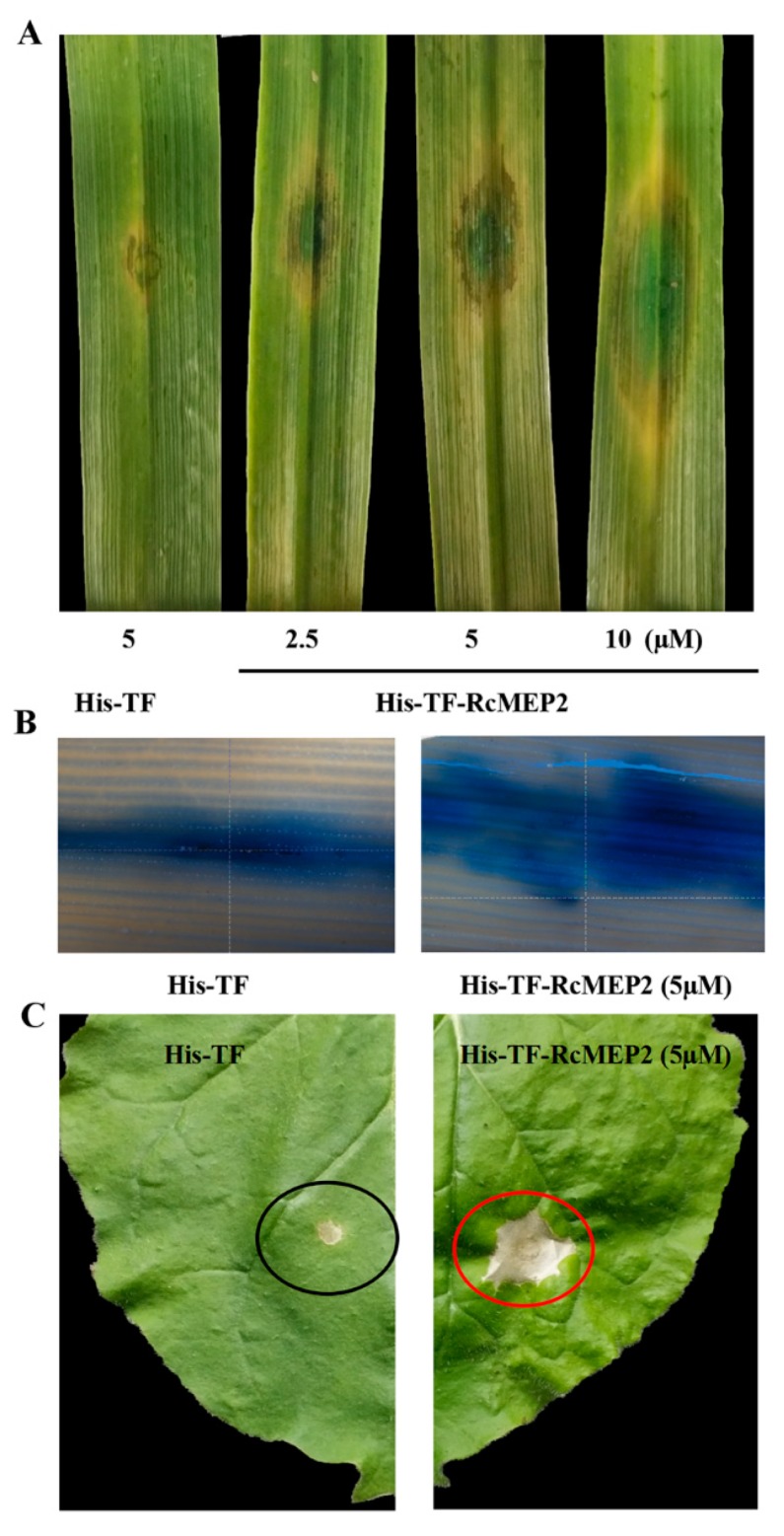
The cell death-inducing activity of RcMEP2 in the protein-infiltrated leaves of wheat and *N. benthamiana*. (**A**) Leaves of a susceptible wheat cultivar Wenmai 6 at three days after infiltration with the His-TF-RcMEP2 (2.5, 5, and 10 µM) or the His-TF-tag solution (5 µM) as control. (**B**) Trypan blue staining of wheat leaves infiltrated with His-TF-RcMEP2 or the control. Dead wheat leaf cells were stained by trypan blue. (**C**) The necrosis on *N. benthamiana* leaves infiltrated with His-TF-RcMEP2 (5 µM). The red and black circles indicate cell death induced by His-TF-RcMEP2 and unobvious cell death with His-TF control, respectively.

To examine if RcMEP2 induces cell death in plants, the purified His-TF-RcMEP2 protein was infiltrated into leaves of a susceptible wheat cultivar Wenmai 6 with concentrations of 2.5, 5, or 10 µM. The results showed that the cell death/necrosis symptoms clearly appeared in the surrounding areas infiltrated by His-TF-RcMEP2 on these wheat leaves at three days after infiltration with all three concentrations (2.5, 5, or 10 µM), whereas the His-TF infiltration (the control, CK) did not cause obvious necrotic symptoms (Figure 4A). With increasing concentration of His-TF-RcMEP2, the necrosis sizes induced by RcMEP2 were elevated (Figure 4A). Trypan blue staining and stereomicroscopy observation results clearly showed that compared with those treated by His-TF, plant cell death stains in the necrosis areas induced by His-TF-RcMEP2 on these wheat leaves were larger and marked (Figure 4B), further illuminating that RcMEP2 treatment induces plant cell death. In addition, we examined the cell death-inducing activity of RcMEP2 in *Nicotiana* *benthamiana* leaves, and similar results were obtained with the RcMEP2; His-TF-RcMEP2 (5 µM) infiltration was able to induce necrosis/cell death, but not with His-TF (CK, 5 µM) (Figure 4C). These results revealed that RcMEP2 was capable to induce the plant cell death. 

**Figure 5:**
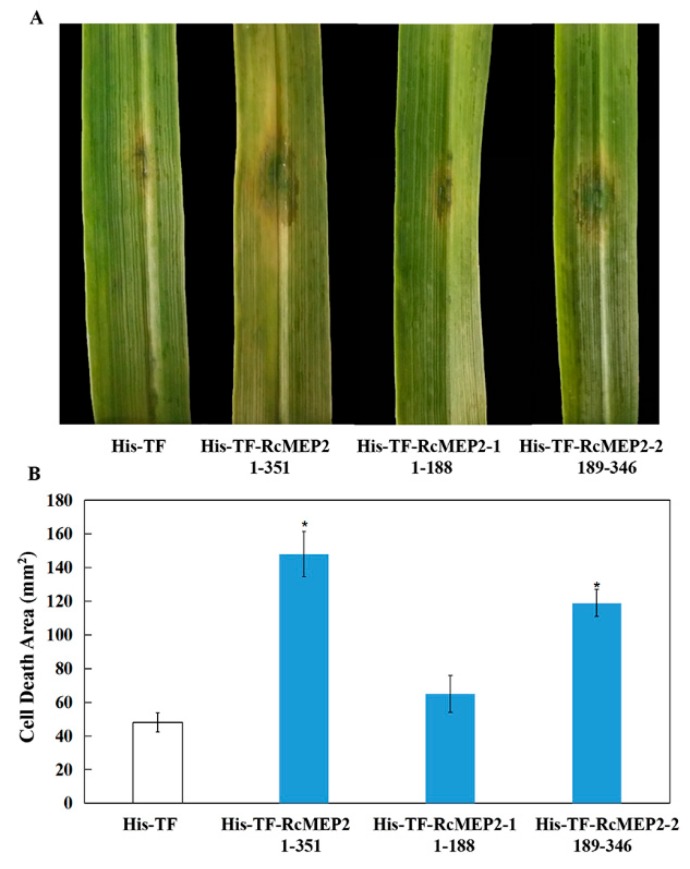
RcMEP2 and its M35 domain can induce cell death/necrosis in infiltrated wheat leaves. (**A**) Detection of the cell death-inducing activities of His-TF-RcMEP2, His-TF-RcMEP2-1, and His-TF-RcMEP2-2 in the infiltrated wheat leaves. (**B**) Areas of necrosis/cell death induced by RcMEP2 and its M35 domain-containing peptides on these wheat leaves. Asterisk * indicates significant difference between His-TF-RcMEP2 or His-TF-RcMEP2-2 treatment and His-TF (control, CK) treatment (*p* < 0.05, *t*-test).

To determine if the M35 domain of RcMEP2, named RcMEP2-2, is required for the cell death-inducing activity, these His-TF-RcMEP2, His-TF-RcMEP2-1, and His-TF-RcMEP2-2 proteins were infiltrated into wheat leaves with the same concentration (5 µM), respectively. The results displayed that similar necrosis sizes were observed with the His-TF-RcMEP2 and His-TF-RcMEP2-2, but His-TF and His-TF-RcMEP2-1 without the M35 domain could not induce obvious necrosis/cell death (Figure 5A,B). These data clearly suggest that this M35 domain is necessary for the cell death-inducing activity of this metalloprotease RcMEP2.

### 2.4. The Signal Peptide is Required for Secretory and Cell Death-Inducing Abilities of the Transiently-Expressing RcMEP2 in N. benthamiana

**Figure 6:**
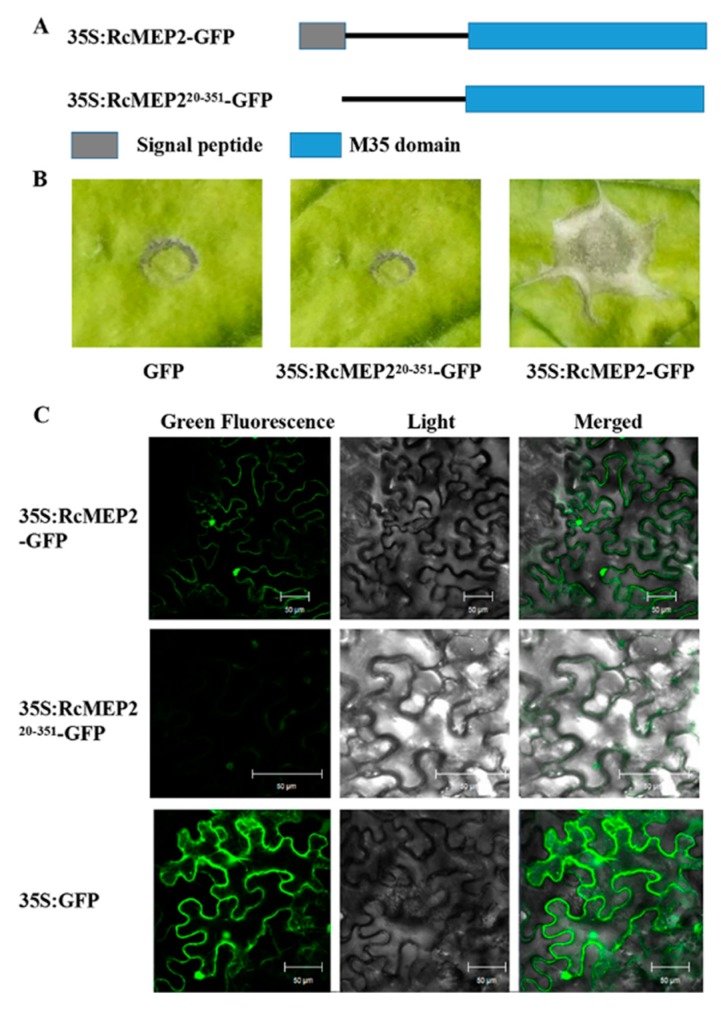
The signal peptide function and cell death-induced activity of RcMEP2 through *Agrobacterium tumefaciens*-mediated transient expression in *N. benthamiana*. (**A**) Scheme of RcMEP2-GFP and RcMEP2’s signal peptide-deleting-green fluorescent protein (GFP) expressing vectors, p35S:RcMEP2-GFP and p35S:RcMEP2^20-351^-GFP. (**B**) The plant cell death symptoms of the leaves agroinfiltrated by the indicated proteins. (**C**) Confocal microscopy images of 35S:RcMEP2-GFP, 35S:RcMEP2^20-351^-GFP, or 35S:GFP in these agroinfiltrated leaves of *N. benthamiana*. These GFP signals were observed under a confocal microscope. Bars = 50 µM.

To examine if the predicted signal peptide functions and is required for the secretion of RcMEP2 to plant apoplasts, and to explore whether *Agrobacterium*-mediated transient expression of RcMEP2 in *N. benthamiana* induces plant cell death, we constructed RcMEP2-green fluorescent protein (GFP) or the signal peptide-deleting-GFP fusion expressing vectors, p35S:RcMEP2-GFP and p35S:RcMEP2^20-351^-GFP (Figure 6A). These fusion proteins were subjected to *Agrobacterium*-mediated transient expression in *N. benthamiana* leaves and the GFP localization was observed and photographed via a confocal microscope. These results exhibited that in these *N. benthamiana* leaves, the transiently-expressing RcMEP2-GFP could induce plant cell death (Figure 6B) and could secrete into the apoplasts (Figure 6C). In contrast, the expressing RcMEP2^20-351^-GFP, lacking the signal peptide of RcMEP2, failed to induce the cell death in *N. benthamiana* and could not obviously secrete into the apoplasts (Figure 6B,C). These results demonstrated that RcMEP2 could act as an apoplastic elicitor of plant cell death, and that the predicted signal peptide of RcMEP2 functioned and it was required for the secretory and cell death-inducing activities of RcMEP2.

### 2.5. RcMEP2 and Its M35 Domain Peptides Contribute to Pathogenicity of R. cerealis to Wheat

**Figure 7:**
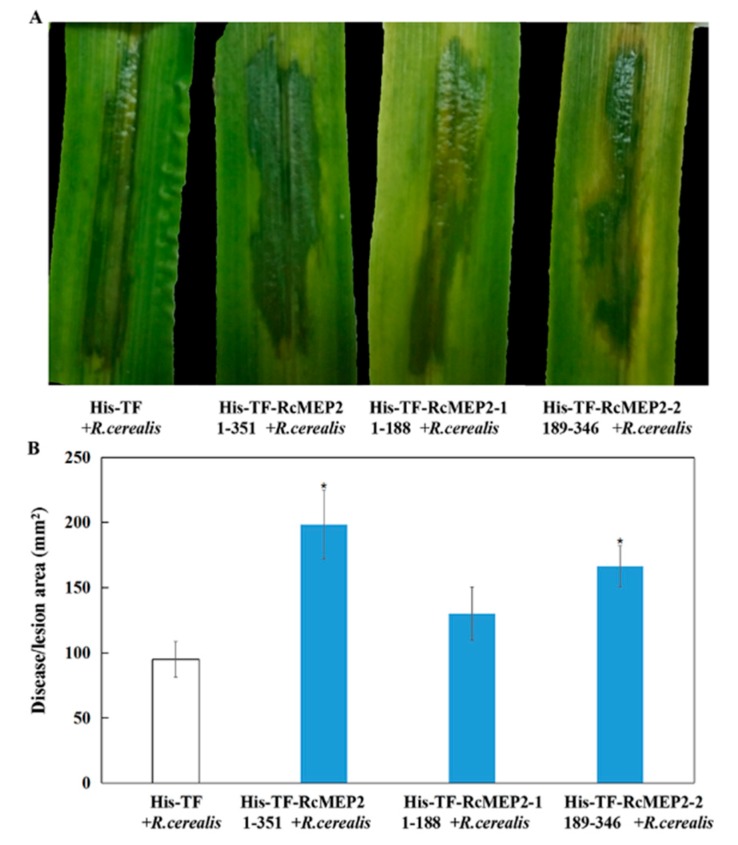
RcMEP2 protein and its M35 domain-containing peptide contribute to pathogenicity of *Rhizoctonia cerealis* to wheat. (**A**) His-TF-RcMEP2 and His-TF-RcMEP2-2 (M35 domain-containing peptide) enhanced pathogenicity symptoms of the invaded *R. cerealis* mycelia in leaves of a wheat cv. Wenmai 6. Photos of the treated leaves were taken at three days post-inoculation with the fungus and the lesion area was measured. (**B**) Disease severity measured as area of lesion induced by *R. cerealis* liquid mycelia on leaves shown in (A). Asterisk * indicates significant difference between His-TF-RcMEP2 or His-TF-RcMEP2-2 treatment and His-TF (CK) treatment (*p* < 0.05, *t*-test).

To further determine the virulence roles of RcMEP2 protein or its M35 domain-containing peptide (RcMEP2-2), His-TF-RcMEP2, His-TF-RcMEP2-1, His-TF-RcMEP2-2, and His-TF were individually infiltrated into leaves of a susceptible wheat cv. Wenmai 6 and then the level surfaces were further inoculated with liquid hypha of *R. cerealis* Rc207. Disease developments were assessed with both time progress and the water-soaking (lesion) area. The results indicated that in comparison with those pre-infiltrated with His-TF-RcMEP2-1 and the His-TF control, the disease development was more rapid, and the disease lesions were larger in leaves pre-infiltrated with both His-TF-RcMEP2 and His-TF-RcMEP2-2 (Figure 7A). The statistical analysis also revealed a significant difference between the His-TF-RcMEP2 and the His-TF control, whereas no significant difference existed between the His-TF-RcMEP2-1 and the His-TF control (Figure 7B). These results clearly suggest that RcMEP2 acts as a virulence factor during *R. cerealis* infection to wheat, and that the M35 domain is required for the virulence of RcMEP2.

### 2.6. RcMEP2 can Promote Accumulation of H_2_O_2_ in Infiltrated Plant Leaves

**Figure 8:**
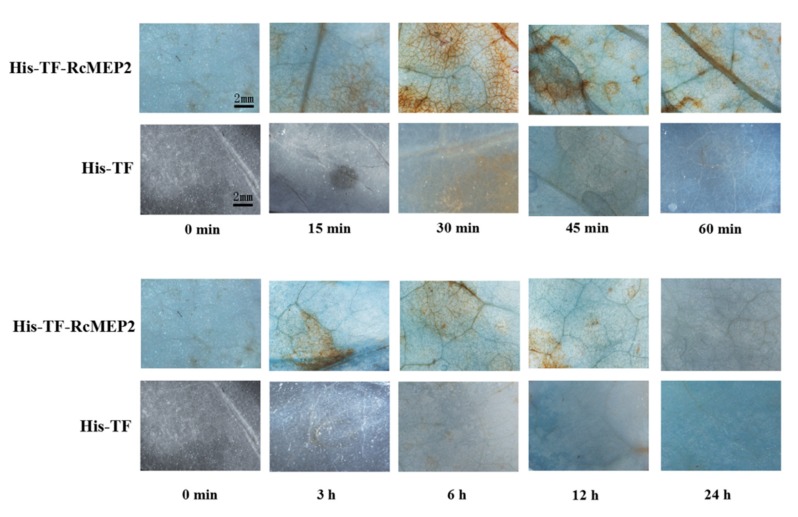
H_2_O_2_ accumulation triggered by RcMEP2 in the infiltrated *N. benthamiana* leaves. H_2_O_2_ accumulation (as indicated by diaminobenzidine staining) appeared in the veins and stomata of His-TF-RcMEP2 infiltrated leaves but not in leaves infiltrated with 5 µM His-TF-tag solution (CK). These stains were observed and photographed under a stereomicroscope. Bar, 2mm.

The excessive accumulation of ROS may promote plant cell death. Hydrogen peroxide (H_2_O_2_) is a major type of ROS. Here, we assayed if His-TF-RcMEP2 protein triggers H_2_O_2_ excessive accumulation when infiltrated in the *N. benthamiana* leaves. The diaminobenzidine (DAB) staining for H_2_O_2_ detection was observed and photographed under a stereomicroscope. As shown in Figure 8, H_2_O_2_ excessive generation was detected from 15 min to 12 h after infiltration with His-TF-RcMEP2, mainly concentrated in the veins and stomata of the treated *N. benthamiana* leaves. The highest accumulation of H_2_O_2_ occurred at 30 min after infiltration, suggesting that the excessive generation of H_2_O_2_ triggered by RcMEP2 is rapid. Nonetheless, no obvious accumulation of H_2_O_2_ appeared in the veins and stomata of His-TF infiltrated leaves (Figure 8). 

### 2.7. RcMEP2 Inhibits the Expression of Chitinases in Wheat

**Figure 9:**
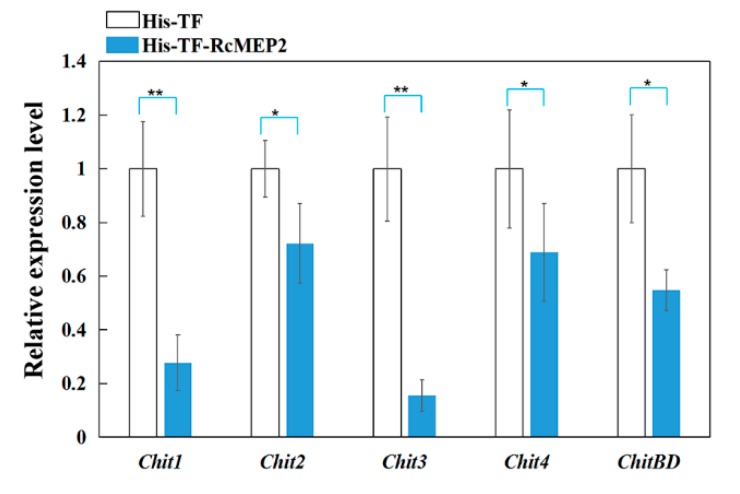
Inhibition of RcMEP2 on the transcription of wheat chitinases. Both samples were derived from the susceptible wheat cv. Wenmai 6 leaves at three days after inoculation with the His-TF-RcMEP2 and the His-TF (CK) at a concentration of 5 µM. The wheat *Actin* gene was used as an internal control to normalize the data. Error bars (SE) were calculated based on three replicates. Asterisk * indicates significant difference (** *p* < 0.01 or * *p* < 0.05, *t*-test) between His-TF-RcMEP1 treatment and His-TF (CK) treatment.

To explore if RcMEP2 treatment inhibits chitinases in wheat, qRT-PCR was deployed to examine transcriptional levels of five kinds of wheat chitinase-coding genes in the susceptible wheat cv. Wenmai 6 leaves infiltrated with His-TF-RcMEP2 or the His-TF control. These tested chitinases include Chit1, Chit2, Chit3, Chit4, and ChitBD of wheat. The results displayed that transcriptional levels of all these five chitinase-encoding genes were significantly decreased in the His-TF-RcMEP2 infiltrated wheat leaves compared to His-TF infiltrated control leaves (Figure 9). Our data suggest that RcMEP2 repressed the expression of all the tested chitinases, supporting previous reports about the inhibiting effect of fungal metalloproteases on host chitinases [27,32,33].

## 3. Discussion

Microbial phytopathogens have developed sophisticated strategies to infect their host plants, such as secreting toxics, pathogen-associated molecular patterns, elicitors, effectors, virulence factors, and metabolites. Meanwhile plants have evolved innate immune systems to protect themselves from invasion of microbial pathogens. For example, chitinases can inhibit growth of many pathogenic fungi through hydrolysis of chitin that is the major structural component of fungal cell walls. Many pathogenic microorganisms can secrete peptidases/proteases including metalloproteases to truncate host chitinases and thereby overcome the deleterious effects of chitinases during their infection process to plants [23,24,25,26,27,28,29,30,32,33,34]. Some M36 family metalloproteases in phytopathogenic fungi have been shown to play key roles in virulence to host plants through degrading host chitinases [27,30,32,33]. However, the virulence roles of M35 family metalloproteases in phytopathogenic fungi are largely unknown. 

In this work, we identified a total of four M35 family metalloproteases from the sequenced *R. cerealis* genome, characterized their gene structures and transcription patterns, and investigated the role of the metalloprotease RcMEP2 in the fungal virulence. RcMEP2, showing the highest transcription-induction at the early infection stages to wheat, was demonstrated to be an apoplastic elicitor of cell death and to act as a virulence factor during the fungus infection to wheat. RcMEP2 and its M35 domain-containing peptides have been evidenced to be able to induce necrosis/cell death and to trigger H_2_O_2_ rapidly-excessive accumulation in the infiltrated leaves of a susceptible wheat cv. Wenmai 6 and *N. benthamiana,* in turn, and to contribute to the pathogenicity of *R. cerealis* to wheat. *Agrobacterium tumefaciens*-mediated transient expression in *N. benthamiana* leaves further indicated that the predicated signal peptide of RcMEP2 functions and is required for the secretion and cell death-induction of this metalloprotease RcMEP2. To our knowledge, this is the first investigation focusing on the virulence role of M35 metalloproteases in *R. cerealis* and this study provides insights on roles of signal peptide and M35 domain of M35 metalloproteases in plant pathogen fungi.

In *R. cerealis* genome, four M35 metalloprotease-encoding genes, *RcMEP2-RcMEP5,* were found. RcMEP2-RcMEP5 proteins are predicted as the secretory M35 family metalloproteases, since their amino acid sequences all contain a signal peptide and an M35 domain that includes the two conserved motifs HEXXE and GTXDXXYG [19,21,35]. Transcriptional levels of *RcMEP2, RcMEP3, RcMEP4*, and *RcMEP5* were quite low during in vitro culturing. In comparison with in vitro culturing, transcriptional levels of *RcMEP2, RcMEP3, RcMEP4,* and *RcMEP5* were sharply induced during the infection process to wheat and reached peaks at the early infection stages (18 or 36 hpi). The markedly induced peaks at the early infection stages imply that these four enzymes might play an important role in fungal pathogenesis. Among them, the transcriptional induction of *RcMEP2* was the highest for all the tested infection time points and peaked at 36 hpi with 162-fold over that of in vitro culturing hypha. The transcriptional inductions of RcMEP3 and RcMEP4 were moderate and peaked at 18 or 36 hpi with 78 or 85-fold over that of in vitro culturing. While transcriptional induction of RcMEP5 was the least and peaked at 18 hpi with 32-fold over that of in vitro culturing. It was reported that the M36 metalloprotease-encoding gene *Cgfl* was strongly upregulated during early infection stages and acted as an effector to enhance virulence in the maize anthracnose fungus *C. graminicola* [27,28]. Additionally, the *R. cerealis* cutinase-encoding gene *RcCUT1* and the *R. cerealis* xylanase-encoding gene *RcXYN1* displayed significant upregulations during the fungus infection process to wheat [11,12]. Taken together, we deduced that RcMEP2 might be the most important one of the four M35 metalloproteases during the fungal infection to wheat, and further investigated the functional role of RcMEP2. 

AsaP1 (MEROPS ID M35.003) is a deuterolysin/M35 metalloprotease in the fish pathogenic bacterium *A. salmonicida* subsp. *Achromogenes,* whose infection can cause significant diseases in a number of fish species and has proven to be an important virulence factor [24]. Another paper reported that NMP1, a neutral M35 metallopeptidase of *Trichoderma guizhouense* NJAU 4742 that can suppress the causative agent of banana wild disease *Fusarium oxysporum* f. sp. *cubense* 4, was required for mycotrophy and self-defense [35]. Our analysis indicate that the RcMEP2 protein sequence contains the M35 metalloprotease-function characteristic motifs HEQSH and GTQDVVYG in the M35 domain. It was reported that the two zinc-binding H residues and a catalytic E residue form the catalytic cluster in the HEXXH motif and the further third zinc ligand (a D residue) is formed by the GTXDXXYG motif C-terminal to the H zinc ligands [21,35]. Interestingly, a recent study reported that for the M36 metalloprotease fungalysin Cgfl in the pathogenic fungus *C. graminicola*, three conserved residues H, E, and H in the HEXXH motif were important in the fungalysin–chitinase interaction [27]. Our results exhibited that RcMEP2, an M35 metalloprotease in *R. cerealis*, significantly inhibited the expression of five kinds of chitinases in infiltrated wheat leaves, but the negative control failed. Thus, we deduce that RcMEP2 may function as a metalloprotease to inhibit chitinase expression and to decrease chitinase activity in the treated host wheat plants. These findings are supported by previous reports about the inhibiting effects of M36 metalloproteases on chitinases [27,28,29,30,32,33]. 

Necrotrophic pathogens thrive on dead host tissues. They often secrete effectors and phytotoxic secondary metabolites, trigger ROS excessive accumulation in host plants, and induce plant cell death, consequently facilitating the growth of necrotrophic pathogens in host plants [10,14,36]. In rice blast fungus *M. oryzae,* Avr-Pita, belonging to the M35 metalloprotease family, acts as an effector and its M35 domain directly interacts with the rice resistance protein Pi-ta, which confers rice blast resistance [26]. In other phytopathogenic fungi, several M36 metalloproteases have been evidenced to be important virulence factors or effectors [27,28,29,30,32,33]. Our previous studies showed that the *R. cerealis* cutinase RcCUT1 and the *R. cerealis* xylanase RcXYN1 could trigger plant cell death and H_2_O_2_ accumulation, and upregulated transcriptions of several defense-related genes in protein-treated wheat plants, leading to enhanced disease [11,12]. Here, sequence analysis showed RcMEP2 might be a secreted protein. Through *A. tumefaciens*-mediated transient expression in *N. benthamiana* leaves, the results showed that the transiently expressed RcMEP2 could secret into the apoplasts of *N. benthamiana* leaves and triggered plant cell death, but the mutant that deleted the signal peptide of RcMEP2 lost these activities. These results demonstrate that RcMEP2 indeed is a secreted protein, and that the predictive signal peptide of RcMEP2 functions and is necessary for secretory and cell death-inducing activity of RcMEP2. Using the purified RcMEP2 protein infiltrated into wheat and *N. benthamiana* leaves, our results reveal that the RcMEP2 is capable of inducing cell death and triggering H_2_O_2_ rapidly-excessive accumulation in the treated leaves. Unlike the induction effect of the cutinase RcCUT1 and xylanase RcXYN1 of *R. cerealis* on transcriptions of several defense-related genes in these protein-treated wheat plants [11,12], RcMEP2 did not upregulate the transcriptions of these host defense-related genes and repressed the expression of wheat chitinases, implying distinct mechanisms underlying the roles of RcMEP2, RcCUT1, and RcXYN1. As expected, RcMEP2 treatment significantly increased disease severity compared to the negative controls, indicating that RcMEP2 treatment enhances virulence of *R. cerealis* during the fungal infection to wheat. Taken together, these results suggest that RcMEP2 acts as a virulence factor and facilitates the infection of the necrotrophic fungal pathogen *R. cerealis* to wheat via triggering H_2_O_2_ rapidly-excessive accumulation and inducing plant cell death as well as inhibiting chitinases in host plants. Moreover, our results indicated that its M35 domain could generate necrosis and disease sizes similar to the full-length RcMEP2, but the M35 domain-deleting peptide RcMEP2-1 could not. Another study also demonstrated that an M35 domain is necessary for the role and interaction of the M35 metalloprotease Avr-Pita in *M. oryzae* with the rice resistance protein Pi-ta [26]. Our results provide solid evidence that the signal peptide and M35 domain are necessary for the functional role of RcMEP2.

In summary, we identified four M35 metalloprotease-encoding genes *RcMEP2-RcMEP5* from *R. cerealis* genome sequences. All the four deduced protein sequences contain a signal peptide and an M35 domain that includes two conserved motifs HEXXE and GTXDXXYG. Although the transcription levels of *RcMEP2-RcMEP5* elevate during the fungal infection process to wheat, the transcriptional induction of RcMEP2 was the highest among these four metalloprotease genes at the early infection stage. RcMEP2 proved to be a virulence factor during the compatible interaction of *R. cerealis* with wheat through triggering H_2_O_2_ rapidly-excessive accumulation, inducing cell death, and inhibiting chitinases in the treated host plants. The signal peptide and its M35 domain are necessary for the functional role of RcMEP2. These findings facilitate a better understanding of pathogenesis mechanism of M35 metalloproteases and infection mechanism of *R. cerealis* to wheat. 

## 4. Materials and Methods

### 4.1. Fungal Isolate, Plant Materials, and Treatments

The necrotrophic fungus *R. cerealis* isolate Rc207 (WK207) is a highly aggressive strain collected in Shandong [3]. A wheat cultivar Wenmai 6, highly-susceptible to *R. cerealis* infection, was provided by Lihui Li at the Institute of Crop Sciences (ICS), Chinese Academy of Agricultural Sciences (CAAS). *Nicotiana benthamiana* was kindly provided by Jianlong Xu at the ICS, CAAS.

The fungus Rc207 was cultured on potato dextrose agar (PDA) for 10 d at 25 °C. The hyphae well-developed on the surface of the PDA were scraped into a 2 mL yeast liquid medium containing steel beads, and the hyphae were completely broken on a vortexer for the pathogenicity test of RcMEP2. A stopper 1 cm^2^ from the edge of the fungal medium was inoculated to the liquid yeast medium containing the toothpick and cultured for ~10 d at room temperature.

Wheat plants were grown in a 13 h light (~23 °C)/11 h darkness (~12 °C) regime. At tillering stage of the wheat cv. Wenmai 6, the bases of the first to second sheaths/stems of the wheat plants were inoculated with small toothpick fragments harboring the well-developed mycelia of *R. cerealis* according to the cultured toothpick method described by Cai [37]. These first to second stems and sheaths were sampled at 18, 36, 72, 96, and 240 h post inoculation (hpi) for RNA extraction and RcMEP2 expression during the infection to wheat plants. *N. benthamiana* was planted in an artificial climate incubator at 25 °C, with a 16 h light / 8 h darkness regime [38].

### 4.2. Identification of M35 Family Metalloproteases in Rhizoctonia Cerealis

The members of candidate M35 family metalloproteases were identified using BLASTP with an E-value less than 1e^−10^ from *R. cerealis* Rc207 genome sequence (unpublished data). The codes indicating the enzyme classes were those defined by the MEROPS [21]. Secreted proteins were identified using three programs that are commonly used to identify protein localization as previously described [39,40]. Secretory status, and transmembrane domains were predicted with SignalP v4.1 (http://www.cbs.dtu.dk/services/SignalP-4.1/), and TMHMM (http://www.cbs.dtu.dk/ services/TMHMM/). Putative extracellular proteins containing signal peptide but lacking a transmembrane domain were identified as secreted proteins. Amino acid sequences used for conserved residues analysis were as indicated. A neighbor-joining tree was constructed using MEGA (version 7.0) program [41].

### 4.3. RNA Extraction, cDNA Synthesis, and All Primers

By using a modified Cetyltrimethyl Ammonium Bromide (CTAB) extraction method [42], total DNA of the fungus was extracted from the hypha of *R. cerealis* 207. Using the TRIzol (Invitrogen, Life Technologies, Carlsbad, CA, USA), according to the manufacturer’s instruction, total RNAs were extracted from the *R. cerealis* Rc207 and the pathogen-inoculated wheat stems/sheaths as well as the His-TF-RcMEP2/His-TF infiltrated wheat leaves. They were subjected to digestion with RNase-free DNase I (Takara, Takara, Japan) and purification [43]. Reverse transcription was performed according to the RNA PCR Kit 3.0 instructions (Takara, Takara, Japan). The SuperScript II First-Strand Synthesis Kit (Invitrogen, Life Technologies, Carlsbad, CA, USA) was used for synthesis of first-strand cDNA. All the primers in the study are listed in Table 1.

### 4.4. Plasmid Construction and Preparation of RcMEP2

Plasmids used were constructed using standard techniques. We amplified full-length RcMEP2 gene sequences from genomic DNA or from the cDNA of *R. cerealis*, depending on predicted introns, using high-fidelity PrimeSTAR HS DNA Polymerase (Takara, Takara, Japan) and using the primers and restriction enzymes listed in Table 1. The *RcMEP2* gene was amplified from the necrotrophic fungus *R. cerealis* isolate Rc207 cDNA and cloned into pCOLD and vector pCAMBIA1305.1-GFP.

### 4.5. Transient Expression of RcMEP2 or Its Signal Peptide-Deleting Proteins in N. benthamiana

In order to investigate whether the signal peptide is required for the RcMEP2’s secreting to plant apoplasts and triggering cell death in *N. benthamiana* cells, the encoding sequence of the RcMEP2 signal peptide (locating on the N-terminal 19 amino acids) using SignalP v4.0 [40] was deleted (RcMEP2^20-351^). These resulting sequences and RcMEP2 sequence without the terminal codon were subcloned and fused to a GFP sequence in a binary vector pCAMBIA1305.1-GFP [44], resulting in the p35S:RcMEP2-GFP and p35S:RcMEP2^20-351^-GFP vectors. These vectors were individually introduced into competent cells of *Agrobacterium* *tumefaciens* strain GV3101 (Biomed, Beijing, China). 

*Agrobacteriumtumefaciens* GV3101 clones carrying p35S:RcMEP35-GFP or p35S:RcMEP2^20-351^-GFP were cultured in Luria-Bertani (LB) medium containing 50 g/mL Kanamycin and 50 g/mL Rifampicin at 28 °C for 48 h, and then centrifuged at 4000 rpm for 10 min at 4 °C to collect the cells. Following dilution with suspension to OD_600_ 1.6–1.8 and placed at 28 °C for 2–3 h, the cell solutions were infiltrated into the leaves of *N. benthamiana* with a l mL syringe without a needle. After infiltration for 3 days, the plant cell deaths triggered by the transient expression proteins in these leaves were observed and photographed [11]. The GFP localization signals were assayed and photographed under a confocal microscope (Zeiss LSM 700, Heidenheim, Germany).

### 4.6. qRT-PCR Analysis

To test transcription profiles of *RcMEP2-RcMEP5* during the fungal infection to wheat, *R. cerealis* Rc207 RNAs were extracted from the inoculated sheaths and stems of wheat plants at different infection time points with *R. cerealis* Rc207 and from *R. cerealis* Rc207 hypha in vitro culturing. To explore if RcMEP2 inhibits chitinases in wheat, qRT-PCR was deployed to examine transcriptional levels of 5 types of chitinase-encoding genes of wheat in the leaves infiltrated with His-TF-RcMEP2 or His-TF (control). These tested wheat chitinase genes include *Chit1* (CK207575.1), *Chit2* (TraesCS6A02G216100.1), *Chit3* (AK446259.1), *Chit4* (AF112966.1), and *ChitBD* (TraesCS2A02G350700.1). The Chit1, Chit2, Chit3, Chit4, and ChitBD sequences were aligned using NCBI. qRT-PCR primers specific to *RcMEP2* and the tested chitinases were designed.

The qRT-PCR was performed using an ABI 7500 RT-PCR system (Applied Biosystems, Waltham, MA, USA) following the modified procedure [45]. In detail, the PCR reaction was performed in 20 μL of reaction mixture: 2× TB Green Premix Ex Taq (Tli RNaseH Plus, TaKaRa, Japan) 10 μL; Primer-F (10 μM) 0.4 μL; Primer-R (10 μM) 0.4 μL; cDNA (diluted 50-fold) 5.0 μL (final concentration ~10 ng); 50× ROX Reference Dye (Tli RNaseH Plus, TaKaRa, Japan) 0.4 μL; RNase-Free ddH_2_O 4.2 μL; and the program used was as follows: 1 min at 94 °C; followed by 40 cycles of 95 °C 5 s, 57 °C 15 s, and 72 °C 34 s. The relative expression levels of target genes in *R. cerealis* or wheat were calculated using the 2^−ΔΔCT^ method [46], where *R. cerealis Actin* gene (*RcActin*) or wheat *Actin* gene (*TaActin*) was used as the internal reference for *RcMEP2* or wheat chitinases. Each RNA sample/primer combination was performed with three independent technique replications.

### 4.7. Heterologous Expression and Purification of RcMEP2 and Its M35 Domain-Containing/Deleting Peptides

The sequence of the *RcMEP2* full-length ORF and its M35 domain-containing/deleting peptide-encoding sequences were sub-cloned into the *Bam*HI site of pCOLD vector (Takara, Takara, Japan), and fused with the His-TF (control) tag in the vector to obtain expression vectors His-TF-RcMEP2 (the whole RcMEP2 protein), His-TF-RcMEP2-1 (its M35 domain-deleting peptide), and His-TF-RcMEP2-2 (its M35 domain-containing peptide), respectively.

The resulting His-TF-RcMEP2, His-TF-RcMEP2-1, and His-TF-RcMEP2-2 fusion constructs were transformed into competent cells of *Escherichia coli* BL21 (DE3), respectively. The recombinant His-TF-RcMEP2, His-TF-RcMEP2-1, and His-TF-RcMEP2-2 proteins were separately expressed after induction with 0.5 mM isopropyl-β-D-thiogalactopyranose at 16 °C for 12 h, and purified using Ni^+^ resin (TransGen Biotech, Beijing, China). Protein purity and molecular weight were determined by using SDS-PAGE according to the method described by Lu et al. [12].

### 4.8. Cell Death-Inducing Activities of RcMEP2 Protein and Its M35-Containing Peptide

Cell death-inducing activity was assayed through infiltrating the heterologously-expressed His-TF-RcMEP2 and the control protein His-TF into the detached young leaves of the wheat cv. Wenmai 6 according to the protocol described previously [47,48]. To determine the suitable concentration required, 25 µL of a serially diluted His-TF-RcMEP2 protein solution (2.5, 5, or 10 µM) was infiltrated into wheat leaves. Subsequently, His-TF-RcMEP2, His-TF-RcMEP2-1, and His-TF-RcMEP2-2 proteins were infiltrated into wheat leaves with the same concentration (5 µM), respectively. His-TF protein was used as the negative control. Protein concentrations were usually measured by the method with bovine serum albumin as a standard protein. 

### 4.9. DAB Staining for Detection of H_2_O_2_

*N. benthamiana* leaves at the 4-leaf stage were cut and then infiltrated with purified His-TF-RcMEP2/His-TF (CK), and then sampled (leaf discs) at 0, 15, 30, and 45 min and 1, 3, 6, 12, and 24 h. According to the previously described method [49], H_2_O_2_ in leaf discs was stained with a solution of 1 mg mL^−1^ DAB-HCl (pH 3.8) at 25 °C under dark for 8 h. Subsequently, these leaf discs were boiled for 5 min in 95 % ethanol [50]. After these leaf discs were decolorized with 95% ethanol for 3 d, H_2_O_2_ accumulation in the leaf discs was photographed under a stereomicroscope (Olympus SZX16, Tokyo, Japan). 

### 4.10. Disease Assay of the Purified RcMEP2 and Its M35-Containing Peptide in Wheat 

In a detached-leaf inoculation assay, fully expanded secondary leaves of the susceptible wheat cv. Wenmai 6 at the tillering stage were infiltrated with 5 µM of His-TF-RcMEP2, His-TF-RcMEP2-1, or His-TF-RcMEP2-2. After these proteins, His-TF-RcMEP2, His-TF-RcMEP2-1, and His-TF-RcMEP2-2 were infiltrated for 6 h, the leaves with the completely absorbed proteins were inoculated with 50 µL mycelium suspension of Rc207. The leaves were then placed in Petri dishes containing filter paper saturated with sterile distilled water and kept under a 16 h day/8 h night regime at 25 °C. Pictures of the lesions were taken 3 days after infiltration with Rc207 mycelium suspension, and the lesion areas were measured and calculated by length × width. Six leaves were used in each experiment and three repeats were done.

## Figures and Tables

**Table 1:** Primers and their sequences.

Primer Name	Sequence (5’-3’)
RcMEP2-F	5’-TCGGCTTGGTTACCCTCTTCTAC-3’
RcMEP2-R	5’-CATCCGGGTAGTAGTTACAAGTAAT-3’
His-RcMEP2-F	5’-GGTACCCTCGAGGGATCCATGTTGTTCTCTGCTCTT-3’
His-RcMEP2-R	5’-AAGCTTGAATTCGGATCCAGATTGAGCGGGGGTGTT-3’
His-RcMEP2-1-F	5’-GGTACCCTCGAGGGATCCATGCGCGCTGCTTTCGCTAC-3’
His-RcMEP2-1-R	5’-AAGCTTGAATTCGGATCCAACATAGGCGACACGTTTGC-3’
His-RcMEP2-2-F	5’-GGTACCCTCGAGGGATCCGGATGCAGCTCAACTCGCCA-3’
His-RcMEP2-2-R	5’-AAGCTTGAATTCGGATCCGTTCTCCGCAAAGTATTCAT-3’
TaActin-F	5’-CACTGGAATGGTCAAGGCTG-3’
TaActin-R	5’-CTCCATGTCATCCCAGTTG-3’
RcActin-F	5’- GCATCCACGAGACCACTTAC-3’
RcActin-R	5’- GCGTCCCGCTGCTCAAGAT-3’
RcMEP2-QF	5’-CAGCGGAAACCTCGTCTCAT-3’
RcMEP2-QR	5’-GGTCCCGATCGACGAAAAGT-3’
RcMEP3-QF	5’-TACGATTGTTACGCGACCCC-3’
RcMEP3-QR	5’-ATGCGCCAATACCAGTCGAA-3’
RcMEP4-QF	5’-GGTTCGGAGCTTGGGATCAA-3’
RcMEP4-QR	5’-GGGCAAGATCTCGACAACCA-3’
RcMEP5-QF	5’-TGAGCACCGTCAAGTCTCAC-3’
RcMEP5-QR	5’-GGAGCACTCCAGAAGACACC-3’
Chit1-QF	5’-GGTAGCACCGACGTCAAGAA-3’
Chit1-QR	5’-CGGCCCGTAGTTGTAGTTGT-3’
Chit2-QF	5’-AAGAACTACTGCGACCCGAC-3’
Chit2-QR	5’-ACGCTGTTCATCCAGTACCA-3’
Chit3-QF	5’-GACGTGGACTACGAGCACTT-3’
Chit3-QR	5’-GTACTGGACGACGGTGTTGG-3’
Chit4-QF	5’-GCAAGTACGGTTATTGCGGG-3’
Chit4-QR	5’-GGAAAAACTGGCGCGTGTAA-3’
ChitBD-QF	5’-CGACGGCAAGAGGGAGATAG-3’
ChitBD-QR	5’-CGCCCCGTAGTTGTAGTTCC-3’
GFP-RcMEP2-F	5’-ACAGCCCAGATCACTAGTATGCGCGCTGCTTTCGCT-3’
GFP-RcMEP2-R	5’-CTTGCTCACCATGGATCCAGATTGAGCGGGGGTGTT-3’
GFP-RcMEP2^20-351^-F	5’-ACCGGTCCCGGGGGATCCGCTCCTGGCCTCACTCTG-3’
GFP-RcMEP2^20-351^-R	5’-CTTGCTCACCATGGATCCAGATTGAGCGGGGGTGTT-3’
