# Correction: Pan et al. Genome-Wide Identification of M35 Family Metalloproteases in *Rhizoctonia cerealis* and Functional Analysis of RcMEP2 as a Virulence Factor during the Fungal Infection to Wheat. *Int. J. Mol. Sci.* 2020, *21*, 2984

**DOI:** 10.3390/ijms22115531

**Published:** 2021-05-24

**Authors:** Lijun Pan, Shengxian Wen, Jinfeng Yu, Lin Lu, Xiuliang Zhu, Zengyan Zhang

**Affiliations:** 1 Institute of Crop Sciences, National Key Facility for Crop Gene Resources and Genetic Improvement, Chinese Academy of Agricultural Sciences, Beijing 100081, China; plj7512@163.com (L.P.); lulin@caas.cn (L.L.); zhuxiuliang@caas.cn (X.Z.); 2 College of Agriculture, Hunan Agricultural University, Changsha 410128, China; wsx8725@hunau.net; 3 College of Plant Protection, Shandong Agricultural University, Taian 271018, China; jfyu@sdau.edu.cn

In the original article, there was a mistake in Figure 8 as published. The picture of His-TF-RcMEP2 at 60 min was confused by L.J.P. because the sample container marker was blurred [1]. Thus, Figure 8 should be replaced with the following figure (Figure 1).

The correction does not change the scientific conclusions of the article in any way. The authors apologize for any confusion this error may have caused to the readers.

## Figures and Tables

**Figure 1:**
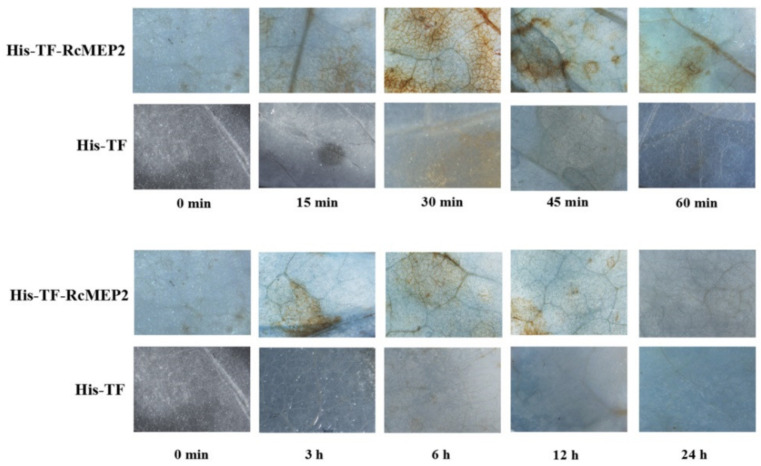
H_2_O_2_ accumulation triggered by RcMEP2 in the infiltrated *N. benthamiana* leaves. H_2_O_2_ accumulation (as indicated by diaminobenzidine staining) appeared obviously in the veins and stomata of His-TF-RcMEP2 infiltrated leaves but not obviously in leaves infiltrated with 5 µM His-TF-tag solution (CK). These stains were observed and photographed under a stereomicroscope. Bar, 2 mm.
